# Emerging roles of haemostatic proteins as markers of disease progression and prognosis in breast cancer

**DOI:** 10.37349/etat.2026.1002361

**Published:** 2026-03-09

**Authors:** Ogochukwu O. Izuegbuna

**Affiliations:** IRCCS Istituto Romagnolo per lo Studio dei Tumori (IRST) “Dino Amadori”, Italy; Department of Haematology and Blood Transfusion, Ladoke Akintola University of Technology (LAUTECH) Teaching Hospital, Ogbomoso 210214, Nigeria

**Keywords:** breast cancer, thrombosis, biomarkers, progression, angiogenesis

## Abstract

Breast cancer is a leading cause of cancer death in women worldwide. One of the major causes of death from breast cancer is metastatic disease, which results from the malignant cells invading and migrating through blood vessels to distant sites. Several studies have shown that metastasis is facilitated by haemostatic proteins. Breast cancer is characterized by a haemostatic imbalance, which is tilted more to a procoagulant state with resultant thrombotic complications. These elements that are involved in thrombosis also play key roles in different aspects of breast cancer growth, including cancer proliferation and progression, cancer survival, angiogenesis, and metastasis. Some of these elements include platelets, endothelial cells, coagulation factors, and fibrinolytic proteins. There is a close relationship between cancer and many of the haemostatic elements. They are usually increased in metastatic breast cancer and have found use as predictive and prognostic markers. Some have been validated in breast cancer. Due to their seemingly active roles in breast cancer progression, some of the haemostatic proteins are being developed as diagnostic tools in the management of breast cancer. They are equally seen as potential targets for the development of novel therapies in breast cancer or repurposing drugs in current use for the same gain. This review highlights the role haemostatic proteins play in breast cancer progression, and their diagnostic and therapeutic relevance.

## Introduction

Breast cancer is the most commonly diagnosed cancer in women worldwide, with more than 2.25 million new cases reported in 2020 [1]. According to GLOBOCAN 2020 data, the mortality rate for breast cancer was approximately 30%, accounting for about 7% of all cancer-related deaths [2]. The majority of these deaths result from metastatic disease [3]. Metastasis is a process involving the complex interplay between intrinsic tumor cell properties and interactions between cancer cells and multiple microenvironments. This leads to the dissemination of cancerous cells to distant tissues and the development of independent secondary masses. Metastasis accounts for an estimated two-thirds of deaths in cancer patients [4], and a population-based study found that more than 50% of patients presenting with breast cancer exhibit osseous metastasis [5].

The process of metastasis is not spontaneous but rather a sequential series of events initiated by the genetic instability of tumor cells due to accumulated mutations. These mutations enable cells to acquire invasive properties, ultimately leading to colonization of new sites [6]. This is a multi-factorial process, as several elements contribute to the sequence of events. While mechanistically complex, research aims to identify genes and proteins involved in the initiation and progression of metastasis, especially in breast cancer patients. These include tumor protein p53, cyclin-dependent kinase inhibitor 2A, phosphatase and tensin homolog, retinoblastoma, hypoxia-inducible factors, and matrix metalloproteinases (MMPs), among others [7–11].

Beyond these factors, the host hemostatic system is increasingly recognized as an important regulator of breast cancer progression. The hemostatic system, which consists of platelets, coagulation, and fibrinolysis pathways, is known to affect different processes in breast cancer progression. Furthermore, aspects of cancer development and progression, such as angiogenesis, immune evasion, anti-apoptosis, and tumor invasion, are strongly associated with the hemostatic system [12–15]. Consequently, venous thromboembolism (VTE) is strongly linked to cancer, with a three to fourfold higher incidence among breast cancer patients compared to age-matched women without cancer [16]. Approximately 50% of cancer patients, and up to 90% of those with metastatic disease, exhibit coagulation abnormalities reflected in laboratory tests. Histopathological analyses show the presence of fibrin and platelet aggregates in and around various tumor types, implying activation of the coagulation cascade. Cancer cells have been shown to express some hemostatic factors and can also endogenously synthesize others [17, 18]. Coagulation factors are known to be in a symbiotic relationship with cancer cells, where they promote each other through metastasis, aiding the hematogenous spread of cancer cells as circulating tumor cells (CTCs) [18].

CTCs are cells from a primary tumor that have entered the vasculature or lymphatics, representing the principal mechanism for the development of metastases. Kirwan et al. [19] demonstrated that the presence of CTCs was significantly associated with increased levels of fibrinogen, D-dimer, thrombin-antithrombin III, and reduced overall survival (OS) in metastatic breast cancer patients. Additionally, CTC-platelet interactions have been shown to transfer the major histocompatibility complex (MHC) to CTCs [20], enabling CTCs to mimic host cells and evade immune detection. Platelets also act as a physical barrier against immune cells [21], stimulate and accelerate epithelial-mesenchymal transition (EMT) in CTCs through the secretion of growth factors [22], and increase CTC adherence to the endothelium through activation of platelet endothelial cell (EC) adhesion molecule-1 (CD31) [23]. Another study showed that high platelet counts were associated with supraclavicular lymph node metastasis and poor prognosis in breast cancer patients [24]. These studies collectively demonstrate the association between the hemostatic system and breast cancer progression.

This review delves into the complex relationship between hemostatic factors and breast cancer, delineating the important roles played by platelets, coagulation, and fibrinolytic proteins in tumor metastasis. It also highlights the potential of targeting hemostatic factors for the management of breast cancer and the prevention of disease progression.

## Overview of hemostasis

The hemostatic system is a complex and delicately controlled physiological system that, under normal circumstances, maintains blood in a fluid state within the circulation. However, it can initiate a cascade of events called coagulation in response to tissue injury to minimize blood loss. Dysfunctional hemostasis can lead to prolonged bleeding if coagulation fails, or to thrombosis if coagulation is excessive. The word “hemostasis” originates from the Greek words “haima” (blood) and “stasis” (to stand still). The process of hemostasis is well-regulated and involves numerous cellular and acellular components. The cellular components include blood cells and vascular system cells, such as red blood cells [25], granulocytes [26, 27], and platelets [28, 29]. The vascular system cells include ECs, smooth muscle, and connective tissue [30], while the acellular components include coagulation factors, the fibrinolysis system, the kinin system, the complement system, and serine protease inhibitors. All these components work in a coordinated manner to arrest bleeding and form a blood clot. The process of hemostasis can be divided into four steps, with the first three primarily involved in clot formation:

A. Vasoconstriction of the blood vessel.
B. Temporary formation of a platelet plug.
C. Activation of the coagulation cascade.
D. Formation of a fibrin plug and fibrinolysis.

Following vascular damage, vasoconstriction of the affected blood vessel occurs almost immediately through stimulation of the sympathetic nervous system of the smooth muscle cells. This is triggered by the release of vasoactive agents such as endothelin-1, bradykinin, vasopressin, and histamine by ECs. This action reduces blood flow by decreasing the vessel diameter, transforming the constitutively anticoagulant endothelium into a procoagulant one to reduce blood loss.

Upon vasoconstriction and endothelial injury, vasoactive agents cause the release of von Willebrand factor (VWF) from the injured site. VWF is a multimeric protein synthesized by ECs and stored in specialized granules called Weibel-Palade bodies. VWF binds through its A3-domain to subendothelial structures, forming a bridge between platelets and ECs. This leads to platelet adhesion to either VWF or exposed subendothelial structures, namely collagen and laminin. During this process, platelets undergo shape changes and release their granules, leading to further platelet recruitment and aggregation and the formation of a temporary platelet plug.

Activated platelets adhere to subendothelial structures and VWF through their glycoprotein (GP) receptors. Adhesion to subendothelial collagen is mediated by two main platelet GPs: GPIa/IIa (integrin α2β1, CD49b/CD29) and GPVI. Platelets also interact with VWF through GPIb/V/IX (CD42a-c), which binds to the A1-domain of VWF. Further platelet-platelet interaction, known as aggregation, is mediated by integrin αIIbβ3 (GPIIb/IIIa). GPIIb/IIIa is the most abundant GP on platelets and plays a critical role in aggregation by binding fibrinogen, which links adjacent platelets to strengthen the platelet plug.

Simultaneously with platelet activation, the coagulation cascade is initiated through two pathways: 1) the extrinsic [tissue factor (TF)] pathway and 2) the intrinsic (contact) pathway. The extrinsic pathway involves the conversion of factor VII (FVII) to activated FVII (FVIIa). This is achieved through activated ECs that release TF (FIII) in response to vascular injury. To prevent inappropriate coagulation initiation, intact ECs express TF pathway inhibitor (TFPI), a serine protease that dampens activation of clotting through the extrinsic pathway by serving as a FXa-dependent inhibitor of the TF-FVIIa complex. However, upon vascular injury, TF—a transmembrane GP found in the adventitial layer of the blood vessel—is exposed to coagulation factors in the blood and directly interacts with FVII. The TF-FVIIa complex activates the zymogens FX and FIX, converting them to FXa and FIXa, respectively. TF acts as a regulatory subunit, while FVIIa catalytically converts FX to FXa or FIX to FIXa. FIXa further enhances the activation of FX, which subsequently converts the zymogen prothrombin (FII) into thrombin (FIIa) in the presence of its cofactor FVa and phosphatidylserine, forming the “prothrombinase complex”.

Coagulation can also occur via the intrinsic pathway when blood is exposed to negatively charged surfaces, leading to the activation of FXII by high molecular weight kininogen (HMWK) and prekallikrein [31]. Auto-activation of FXII also occurs upon contact with substances such as polyphosphates, nucleic acids, heparin, collagen, misfolded proteins, extracellular traps, and anionic bacterial surfaces [32–34]. Prekallikrein can be directly activated by FXII to kallikrein, which in turn activates more FXII to FXIIa, creating a positive feedback loop—the “intrinsic tenase” complex. FXIIa activates FXI to FXIa. HMWK is involved in these steps and is also converted to kinins, which can cause vasodilation, erythema, and pain. FXIa subsequently activates FIX to FIXa with calcium ions as a cofactor. FXIa, together with FVIIIa, phospholipids, and calcium ions—forming the “intrinsic tenase complex”—converts FX to FXa.

The thrombin generated by the prothrombinase complex cleaves fibrinogen to form fibrin. Fibrin polymerization then occurs, building up fibrin monomers into a polymeric mesh that integrates with aggregating platelets to form a clot. To strengthen this fibrin mesh, FXIII (fibrin-stabilizing factor), a transglutaminase enzyme activated by thrombin, forms gamma-glutamyl-lysyl amide cross-links within the fibrin clot, stabilizing it against shear stress. Simultaneously, thrombin activates thrombin-activatable fibrinolysis inhibitor (TAFI), which protects the newly formed fibrin clot from being lysed by fibrinolytic proteins.

Thrombin is central to many actions in the coagulation process; hence, excess thrombin can induce a pathological thrombotic state [35]. Therefore, several anticoagulant mechanisms regulate thrombin generation, including antithrombin, protein C inhibitor, heparin cofactor II, protease nexin 1, cartilage oligomeric matrix protein, and TFPI [36–38].

**Figure 1:**
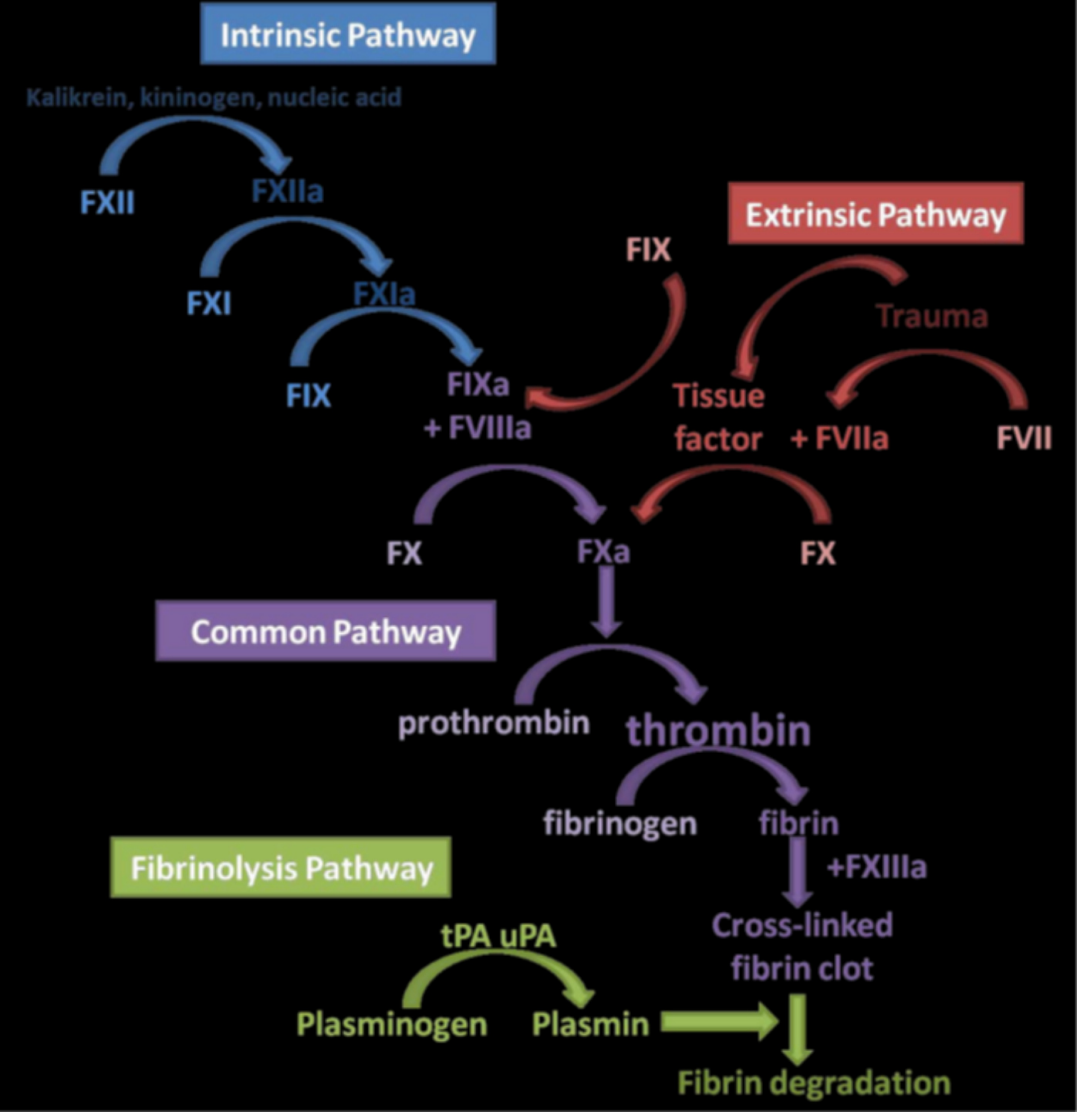
**An overview of the coagulation cascade showing the various haemostatic pathways involved in haemostasis.** FXII: factor XII; tPA: tissue-type plasminogen activator; uPA: urokinase-type plasminogen activator.

The final process of hemostasis, fibrinolysis, is initiated by the release of tissue-type plasminogen activator (tPA) and urokinase-type plasminogen activator (uPA), which convert plasminogen into plasmin. Plasmin then initiates fibrin lysis, leading to the formation of fibrin degradation products (FDPs). However, fibrinolytic inhibitors such as plasminogen activator inhibitor-1 (PAI-1), alpha 2-antiplasmin, and TAFI (which increases resistance to fibrinolysis by tPA) can impede this activity [39] (Figure 1).

## Breast cancer and hemostasis

Like other cancers, breast cancer induces an acquired hypercoagulable state characterized by abnormal laboratory results, reflecting enhanced activation of the coagulation cascade and platelets. Beyond coagulation activation, this state also involves the failure of physiological anticoagulant mechanisms and loss of vascular endothelial integrity due to tumor cells. Evidence for this abounds: histopathological analyses show platelet aggregates and fibrin deposits in and around various tumor types, indicating local activation of blood coagulation. Between 60% and 100% of cancer patients exhibit hemostatic alterations without accompanying thrombotic events [40]. The interaction between cancer cells, the vascular endothelium, and hemostatic proteins is primarily responsible for pro-thrombotic and pro-inflammatory events in cancer. These hemostatic alterations create a crosstalk between thrombus formation and inflammatory responses. For example, fibrin acts as a scaffold for platelets and leukocytes to bind, release inflammatory signals, and play a role in wound healing [41]. Fibrin(ogen) has also been reported to induce the production of proinflammatory signaling molecules by leukocytes [42–44]. Thus, elevated coagulation factors and inflammatory markers are risk factors for cancer-associated thrombosis [45].

This hypercoagulable state is known to influence cancer biology. In breast cancer, hemostatic activation has been shown to be more than just an effect of cancer progression; it is also a major regulator of malignant transformation, tumor angiogenesis, and metastasis [46]. Fibrin provides a scaffold for tumor cell anchorage and invasion, thereby protecting cancer cells from immune recognition. Thrombin has been shown to promote invasive growth and metastasis of cancer cells [47, 48]. D-dimer, the end-product of fibrinogen hydrolysis, is also associated with tumor progression in various cancers, including breast cancer [49, 50]. Thus, the hemostatic alterations associated with breast cancer progression, as with other cancers, manifest in different forms, including prolonged or shortened prothrombin time and activated partial thromboplastin time, increased or decreased levels of thrombin and other coagulation proteins, and thrombocytosis. These alterations have been closely associated with tumor burden and distant metastasis. This section will therefore examine different components of the hemostatic system, discuss their functions, and highlight the important role each plays in breast cancer progression.

## Platelets and breast cancer

Although they are not proteins, platelets are anucleate, discoid-shaped cells measuring between 1.5 and 3 μm in size. As mentioned earlier, they play an important role in vascular hemostasis and are also involved in inflammation, sepsis, wound healing, and immunity. Their primary physiological role is to prevent or stop bleeding through clot formation after activation. However, in pathological conditions like cancer, this role can become dysregulated. Evidence has shown that platelets contribute to breast cancer progression [51, 52]. One such piece of evidence is thrombocytosis, which has been associated with solid tumors for more than a century. Platelet count has also been closely associated with tumor size and stage in breast cancer [53]. Gasic et al. [54] used an animal model to demonstrate a 50% reduction in tumor metastasis after experimental thrombocytopenia induced with neuraminidase and anti-platelet serum, which was reversed by the introduction of platelet-rich plasma. Thrombocytosis is thus recognized as a poor prognostic factor in breast cancer patients [55].

**Figure 2:**
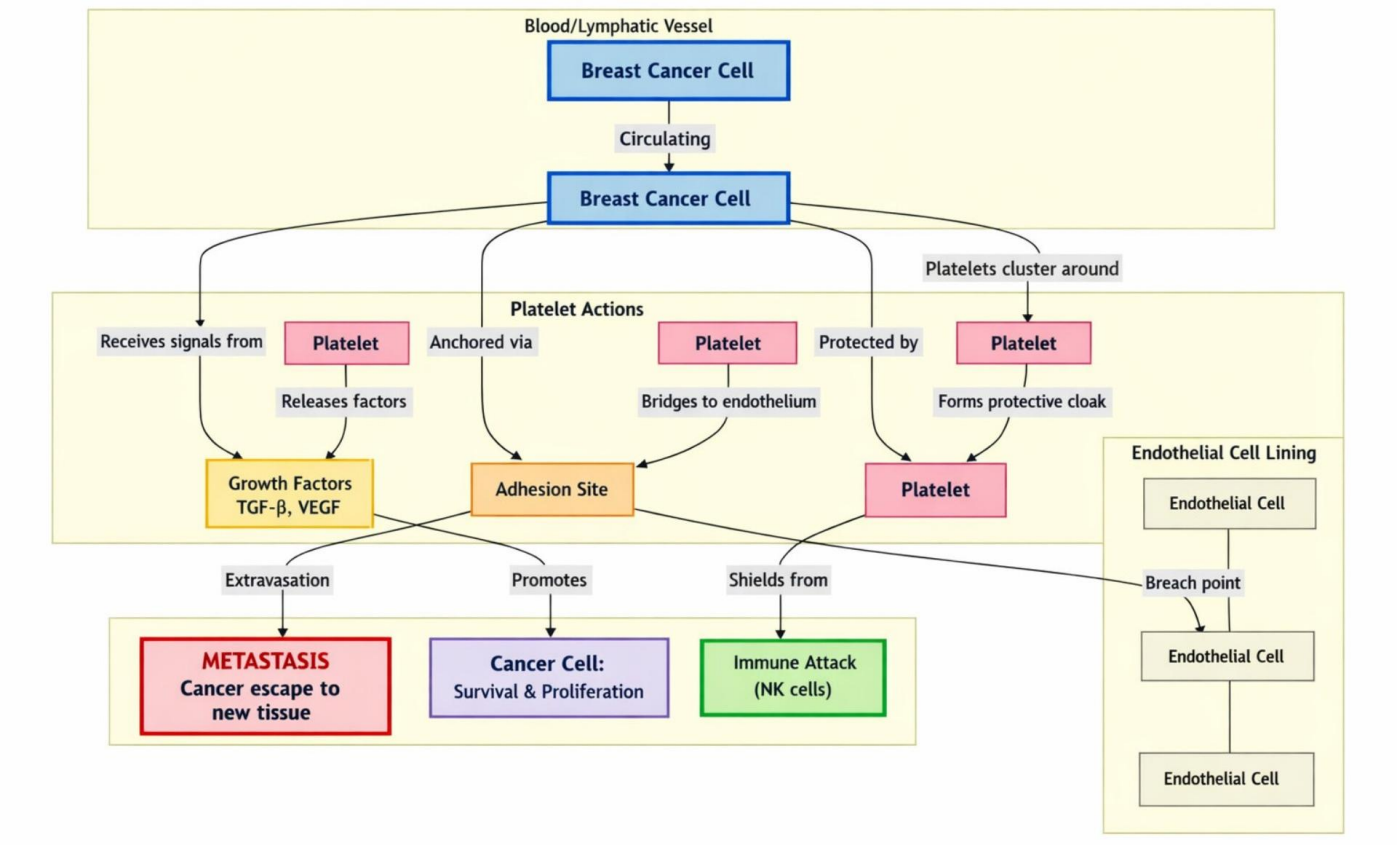
**Platelets promote breast cancer progression through: 1) platelet-derived TGF-β and VEGF, which promote breast cancer proliferation and formation of new blood vessels; 2) cancer cells; 3) physically shielding breast cancer from immune cells like the NK cells.** NK: natural killer; TGF-β: transforming growth factor-β; VEGF: vascular endothelial growth factor.

Platelets promote breast cancer progression and metastasis through various mechanisms, including the promotion of tumor angiogenesis and the facilitation of tumor extravasation via EMT [56]. They also increase the survival of tumor cells in circulation by aiding in immune evasion and phagocytosis avoidance [57] (Figure 2). Under physiological conditions, platelet activation by a stimulus leads to shape change and degranulation. The platelets’ α-granules and dense granules then release various substances that affect homeostasis. Tumor cells exploit this through a process called tumor cell-induced platelet aggregation (TCIPA) [58]. This can occur via tumor cell-induced thrombin generation or through the release of several mediators from activated platelets that induce platelet aggregation and other vascular changes promoting the growth, survival, motility, and extravasation of circulating cancer cells. These mediators include adenosine diphosphate and thromboxane A2, which have been shown to induce platelet aggregation in vitro [59, 60].

**Figure 3:**
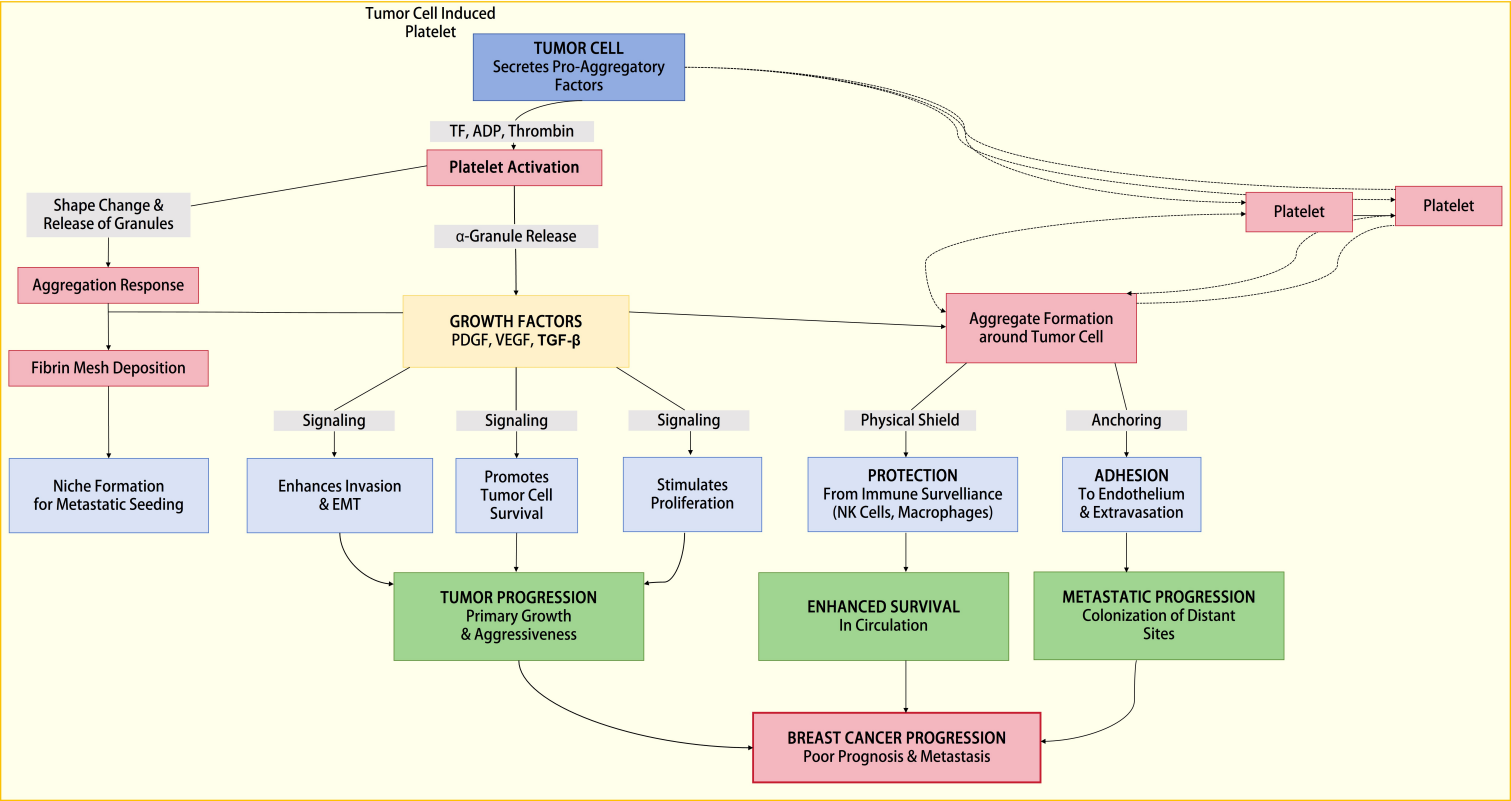
**A representation of the process through which TCIPA promotes breast cancer proliferation and metastasis.** EMT: epithelial-mesenchymal transition; NK: natural killer; PDGF: platelet-derived growth factor; TCIPA: tumor cell-induced platelet aggregation; TF: tissue factor; TGF-β: transforming growth factor-β; VEGF: vascular endothelial growth factor.

Breast cancer cells can also stimulate platelet aggregation via thrombin generation. The thrombin generated activates platelets through protease-activated receptors (PARs) on platelets; PAR-1 to -4, with PAR-1 being the most potent receptor for thrombin. PAR-1 is also observed to be elevated in breast cancer and is associated with cancer progression and poor prognosis [61]. Thrombin-activated platelets express substances that facilitate contact with tumor cells and, in turn, enhance TCIPA. TCIPA is also enhanced via the activation of MMP 2 at the cell surface, mediated by membrane type-1 MMP. This interaction usually involves integrin αvβ3, and the absence of this integrin can reduce platelet aggregation and platelet-cancer cell interaction [62]. These activated platelets can release more substances that play a role in breast cancer metastasis (Figure 3).

One such role is in angiogenesis, an important event in the initiation and metastasis of cancer cells. A reduction in blood supply is known to retards tumor cell growth and can eventually lead to cell death. The process of tumor angiogenesis in breast cancer is a complex interplay between tumor cells and the tumor microenvironment, involving the secretion of various growth and angiogenic factors, many of which are present in the α-granules of platelets. These include platelet-derived growth factor (PDGF), vascular endothelial growth factor (VEGF), transforming growth factor-β (TGF-β), and epidermal growth factor (EGF) [63]. Elevated levels of VEGF activate the VEGF pathway, causing the formation of new blood vessels and breast cancer cell proliferation [64]. The release of angiogenic factors from platelets is usually inflammation-driven, and in breast cancer, may involve inflammatory markers synthesized by cancer cells, such as interleukin-6. The process of VEGF release from platelets has been observed in breast cancer patients; moreover, platelets from breast cancer patients release significantly more VEGF than those from healthy controls, and this release correlates with serum VEGF levels [65, 66]. The platelet pool of VEGF is reported to constitute more than 80% of total circulating VEGF in healthy subjects as well as in breast cancer patients [67]. As tumor cells circulate in the bloodstream, they are shielded by platelets, which at some point release VEGF to induce endothelial wall permeability and angiogenesis, thereby aiding cancer metastasis. Experimental work in breast cancer shows that platelet angiogenic activities are mediated by VEGF—integrin cooperative signaling via the phosphatidylinositol 3-kinase (PI3K)/PKC pathway [68].

Apart from pro-angiogenic proteins like VEGF, platelets also store anti-angiogenic proteins (Table 1), including endostatin, platelet FIV, thrombospondin-1, and PAI-1. These pro- and anti-angiogenic proteins are stored in different compartments of the α-granules and are selectively released upon different stimuli [68, 69]. Both angiostatin and endostatin have been shown to exhibit anti-tumoral efficacy [70]. Additionally, platelets have been shown to regulate vascular integrity and prevent tumor bleeding, especially in murine models of breast cancer [71].

**Table 1:** Some of the granule contents of platelets are involved in angiogenesis.

**Granule type (platelets)**	**Content**	**Role in angiogenesis**
α-Granules (growth factors)	1. Platelet-derived growth factor	Promote
	2. Vascular endothelial growth factor	Promote
	3. Basic fibroblast growth factor	Promote
	4. Epidermal growth factor	Promote
	5. Interleukin-1beta	Promote
	6. Stromal cell-derived factor-1 α	Promote
	7. Angiopoietins	Suppress
	8. Platelet factor IV	Suppress
	9. Endostatins	Suppress
	10. Thrombospondin-1	Suppress
	11. Sphingosine-1-phosphate	Suppress
	12. Tissue inhibitors of metalloproteinases (TIMPs; TIMP 1, 2, and 4)	Suppress
	13. Matrix metalloproteinases (MMPs) (MMP 1, 2, 3, 9, and 14)	Suppress
	14. Hepatocyte growth factor	Suppress
	15. Transforming growth factor-β	Suppress
α-Granules (haemostaticfactors)	1. Factor V	Promote
	2. Von Willebrand factor	Promote
	3. Fibrinogen	Promote
	4. Factor XIII	Promote
	5. Plasminogen	Promote
	6. Tissue factor pathway inhibitor	Suppress
	7. Antithrombin	Suppress
	8. Protein S	Suppress
	9. Plasminogen activator inhibitor-1	Promote
Dense granules	1. Adenosine triphosphate	Promote
	2. Adenosine diphosphate	Promote
	3. Guanosine 5’-triphosphate	Promote
	4. Guanosine diphosphate	Promote

While in circulation, CTCs can be targeted and destroyed by immune cells; however, platelet-tumor cell interaction helps protect some CTCs from natural killer (NK) cell-induced cell death. Platelets prevent CTC death through several mechanisms: 1) interaction between breast cancer cells and platelets has been shown to activate the TGF-β signaling pathway, which promotes metastasis and causes immunosuppression by downregulating DNAM-1 (CD226) and TACTILE (CD96) expression in NK cells [57, 72], 2) breast cancer cells can also ‘pick up’ MHC class I antigens from platelets, helping them evade NK cell recognition as foreign cells, thereby limiting NK cell cytotoxicity [73], 3) platelets have been shown to cleave ligands such as NK group 2, member D from tumor cells’ surface, which prevents NK cell recognition and subsequent cytotoxicity [74], 4) another mechanism involves platelets binding to mucins on the surface of cancer cells through the GPIb-IX-V and GPIIb-IIIa adhesion molecules, mediated by surface integrins αvβ3 or P-selectin on the cancer cells. This can create a physical barrier that prevents NK cells from destroying the cancer cells [75].

Platelets are involved in clot formation through their interaction with the endothelium. This interaction can also facilitate tumor cell extravasation to other sites. GPIIb/IIIa and P-selectin play major roles in platelet-tumor cell adhesion. Zhao et al. [76] demonstrated that GPIIb/IIIa was important for the hematogenous metastasis of human breast carcinoma MDA-MB-231 cells. Research data have also shown that platelet-tumor cell interaction through P-selectin mediates tumor extravasation in a manner similar to leukocyte diapedesis with ECs [77]. Other receptors, like GPIb/IX, have also been implicated in metastasis through VWF-mediated adhesion [78, 79].

Given the established evidence of platelet involvement in breast cancer and other cancer metastases, there has been interest in using antiplatelet therapies to manage cancer progression. However, results have been mixed. A recent phase III trial (NCT02927249) of aspirin use in breast cancer survivors showed no benefit, and thus aspirin was not recommended for breast cancer prevention [80]. Conversely, a recent study of women with inflammatory breast cancer showed that aspirin use was associated with benefits in both OS and disease-free survival (DFS) [81]. Kononczuk et al. [82] demonstrated that abciximab and eptifibatide (GPIIb-IIIa inhibitors) can induce apoptosis in MCF-7 breast cancer cells. These effects, including the prevention of hematogenous metastasis, have been demonstrated in other cancer cell lines as well.

Due to their close interaction with tumor cells, particularly in the tumor microenvironment, platelets have also been investigated as potential drug delivery systems. Yap et al. [83] conjugated monomethyl auristatin E (MMAE), a microtubule inhibitor, to a single-chain antibody targeting platelet GPIIb-IIIa. The antibody-drug conjugate (ADC) successfully targeted MDA-MB-231 tumors in a murine model, leading to a significant decrease in tumor growth compared to untreated mice and mice treated with MMAE conjugated to a non-binding antibody control [83]. This and similar preclinical works have shown the role platelets play in breast cancer progression and suggest they can be exploited for therapeutic benefit [84, 85].

## The tissue factor pathway and breast cancer

TF is a 47 kDa integral membrane GP that initiates the extrinsic pathway by forming a complex with FVIIa [86]. TF acts as the regulatory subunit of the TF-FVIIa complex, while the FVIIa serine protease domain is involved in activating downstream coagulation factors, especially FX [87]. Beyond its hemostatic function, the TF-FVIIa complex can also activate inflammatory pathways by binding to PAR-1 and PAR-2, which play roles in angiogenesis and tumor invasion [88]. The TF gene, located on chromosome 1p21–p22, consists of a 219-amino-acid extracellular domain, a 23-amino-acid transmembrane segment, and a 21-amino-acid cytoplasmic tail. It is regulated by several transcription factors associated with hypoxia and inflammation, including nuclear factor-κB and activator protein. TF is constitutively expressed in subendothelial cells and only interacts with blood upon loss of vascular integrity [89].

While the TF/FVIIa/FXa complex is a recognized player in vascular hemostasis, several studies have shown it plays a major role in cancer progression, especially in breast cancer [90–94]. It is also associated with poor survival across many cancer types [90, 95]. However, Stämpfli et al. [96] did not find an association between TF and survival in breast cancer patients.

As mentioned, TF can activate PARs. The PARs are a four-member subfamily of G protein-coupled receptors (GPCRs) activated by proteolytic cleavage of the extracellular N-terminus. They are involved in hemostasis, angiogenesis, inflammation, neural tube closure, and cell growth [86]. Both the TF-FVIIa complex and the TF/FVIIa/FXa complex mediate TF signaling [97]. Although PARs are primarily thrombin receptors, the TF/FVIIa/FXa complex can also cleave and activate PAR-1; PAR-1 is also cleaved by MMP 1, plasmin, and activated protein C (APC) [97]. PAR-2 is not directly activated by thrombin [98]. The TF/FVIIa and TF/FVIIa/FXa complexes can directly cleave it. The TF-FVIIa complex can trigger a transient increase in calcium, leading to the activation of the mitogen-activated protein kinases (MAPKs) pathway, including p44/42, p38, and c-Jun N-terminal kinase, which play a major role in cell cycle control [99]. It also activates Src-like kinases, PI3K, the Janus-associated kinase (JAK)/signal transducer and activator of transcription (STAT) pathway, and the Rho GTPases Rac1 and Cdc42, which are involved in cell survival and cytoskeletal rearrangements [91, 100]. Beyond the MAPK, PI3K, and JAK/STAT pathways, the TF-FVIIa complex also targets receptor tyrosine kinase (RTK), insulin-like growth factor 1, and integrin signaling. TF association with integrins has been shown to play a role in breast cancer metastasis and cell survival [101, 102]. Breast cancer cells have also been shown to inhibit apoptosis via phosphorylation of the p44/42 MAPK and Akt/protein kinase B signaling through the TF-FVIIa-FXa complex (rather than thrombin), activating the mammalian target of rapamycin (mTOR) pathway, which results in cell migration—an important step in metastasis [103]. Inhibition of this mTOR pathway significantly reduced cell migration [104, 105]. In a similar experiment, simvastatin abrogated TF/FVIIa and TF/FVIIa/FXa complex signaling, inhibiting the phosphorylation of Akt/protein kinase B in a breast cancer cell line and preventing cell proliferation [106]. Through these pathways, TF is involved in breast cancer metastasis, cell proliferation, and survival. Tumor-expressed FVII is also reported to enhance tumor growth and metastasis in breast cancer [107].

TF signaling induces angiogenesis through either clotting-dependent or clotting-independent mechanisms. The clotting-independent mechanism involves PAR-2 signaling, typically achieved through the cytoplasmic domain of TF [108]. The clotting-dependent mechanism involves TF-induced thrombin formation and deposition of cross-linked fibrin, which creates a pro-angiogenic template that aids blood vessel infiltration [108]. TF overexpression has also been shown to enhance cancer cell growth by increasing VEGF transcription and reducing thrombospondin transcription [109]. VEGF is reported to mediate TF expression via early growth response protein 1 [110].

TF exists in three isoforms: full-length TF (as described above), an alternatively spliced TF (asTF) isoform generated by the omission of exon 5 during processing of the TF primary transcript, which causes a frameshift. The asTF protein has a unique C-terminus that lacks a transmembrane domain, making it soluble [111]; and the alternative exon1A-TF (TF-A), produced by alternative splicing involving the first intron. While not particularly pertinent to hemostasis, asTF has been shown to be produced by various cancers, including breast cancer cell lines [112]. The asTF isoform promotes breast cancer progression, metastasis, and tumor angiogenesis, often in a beta integrin-dependent manner [113, 114].

Due to its high expression in different cancer cells and its multiple roles, TF has been proposed as a target for cancer therapy. This includes the use of various antibody technologies that have yielded promising results in different settings [115, 116]. The recent approval of the first TF-targeted ADC, tisotumab vedotin (TV) (HuMax^®^-TF-ADC), for cervical cancer [117] has provided hope for using antibodies against TF in breast cancer. The phase 2 clinical trial NCT03485209 is currently evaluating patients with solid tumors treated with tisotumab in combination with other antineoplastic agents, although breast cancer is not included [118, 119]. Since TF acts mainly through PARs, blocking PAR signaling has also been explored as a therapeutic strategy. Atopaxar and vorapaxar, two PAR-1 inhibitors, have been shown to inhibit cancer progression in preclinical models [120]. The monoclonal antibody Mab5G9, which blocks TF-VIIa-mediated activation of PAR-2 and disrupts the interaction of TF with integrins, has also shown antineoplastic activity in breast cancer preclinical models [121]. Whether these will prove efficacious in breast cancer patients remains to be determined.

FVII and FX are vitamin K-dependent serine proteases; therefore, targeting them with anticoagulants is another therapeutic strategy. Vitamin K antagonists like warfarin have shown antitumor potential through anti-angiogenesis, anti-adhesion, and decreased cell mobility [122, 123]. A FXa inhibitor, rivaroxaban, was assessed for its antineoplastic properties in a phase 2 clinical trial with breast cancer patients (EudraCT 2014-004909-33), but no results are available [124]. However, rivaroxaban has been shown to promote antitumor immunity by enhancing the infiltration of dendritic cells and cytotoxic T cells at the tumor site, blocking FXa PAR-2 signaling [125]. Amblyomin-X, a Kunitz-type FXa inhibitor discovered through transcriptome analysis of the salivary gland from the *Amblyomma sculptum* tick, has also shown potential antineoplastic activity [126]. Utilizing the TFPI pathway, a natural inhibitor of TF, is also believed to be an anticancer strategy for breast cancer progression [127]. Overall, TF signaling, a marker of breast cancer progression, represents a promising target for developing breast cancer treatments.

## Thrombin and breast cancer

Thrombin is a Na^+^-activated allosteric serine protease of the chymotrypsin family, derived from its inactive zymogen prothrombin, a 70 kDa GP synthesized in the liver and secreted into the blood [128]. Once generated by proteolytic cleavage of prothrombin by FXa, it plays two important roles. It acts as a procoagulant by activating fibrinogen to fibrin and activating FV, FVIII, FXI, and FXIII to FVa, FVIIIa, FXIa, and FXIIIa, respectively. It also inhibits fibrinolysis via TAFI activation and activates platelets through PAR-1. Conversely, it acts as an anticoagulant when bound to thrombomodulin, a receptor on EC membranes. This binding suppresses its ability to cleave fibrinogen and PAR-1 but enhances its specificity towards the zymogen protein C, converting it to APC [129]. Thrombin generation is a key step in blood coagulation. Thrombin acts by cleaving PAR-1, PAR-3, and PAR-4 at a specific site within the extracellular N-terminus, exposing a tethered ligand that subsequently folds back to activate the receptor [130]. Cleavage of PAR-1 and PAR-4 leads to platelet activation and aggregation. PAR-3 is not present on human platelets but is widely expressed in other cell types [128].

**Figure 4:**
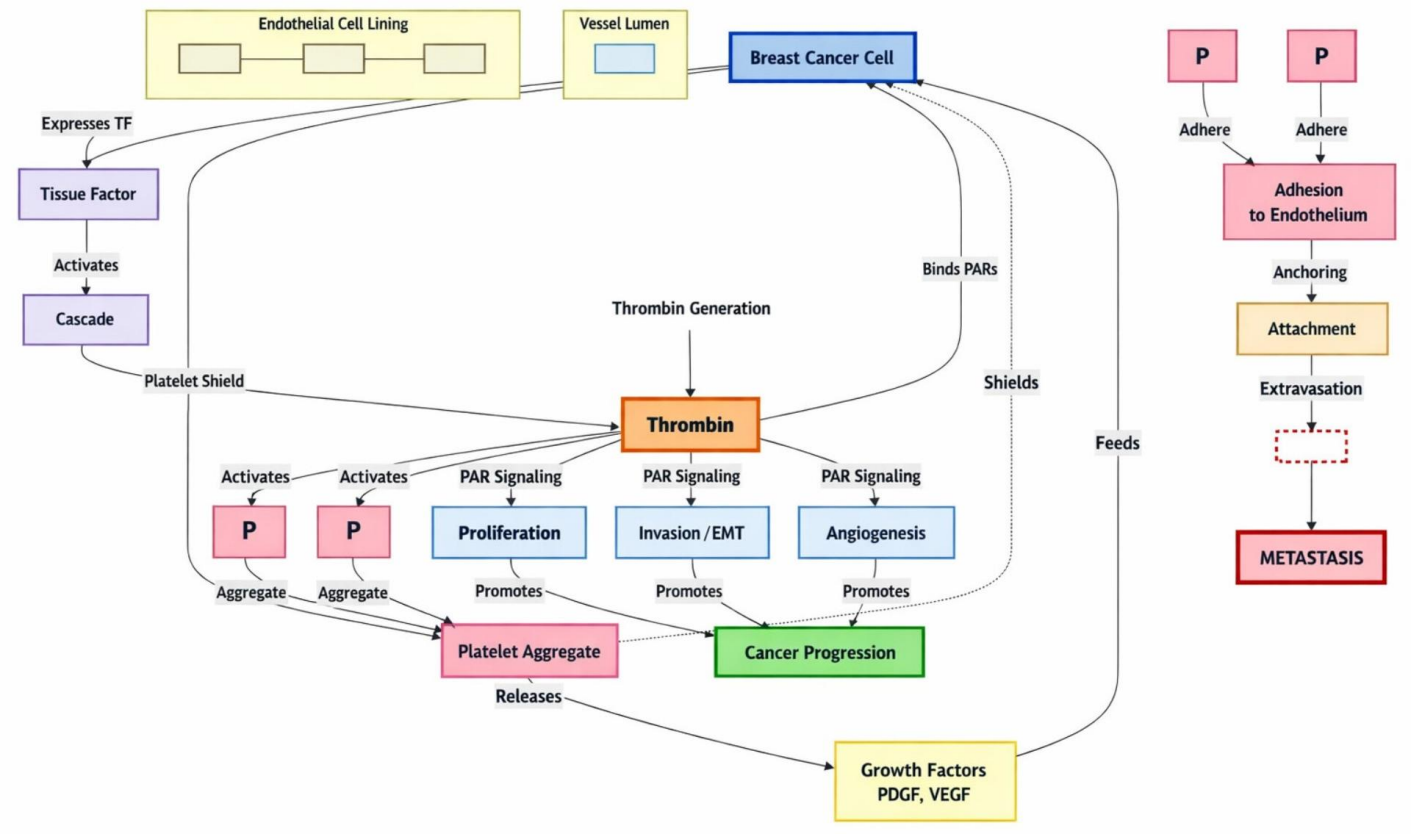
**The various roles thrombin plays in breast cancer proliferation and metastasis.** EMT: epithelial-mesenchymal transition; PAR: protease-activated receptor; PDGF: platelet-derived growth factor; TF: tissue factor; VEGF: vascular endothelial growth factor.

Several studies have shown that thrombin is involved in tumor growth, metastasis, and angiogenesis in different cancer models [131]. It is also reported to increase breast cancer invasiveness. In the prospective HYPERCAN study, thrombin was shown to predict early recurrence in breast cancer, implying its role in tumor growth [132, 133]. PAR-1, the primary receptor for thrombin, is involved in cancer cell invasion and metastasis in multiple cell lines, including breast cancer, and is overexpressed in breast cancer. Wang et al. [134] showed that Twist-mediated induction of PAR-1 can promote EMT, tumorigenicity, and metastasis by controlling the Hippo pathway. This has made PAR-1 a potential therapeutic target in cancer. In preclinical studies of breast cancer cell lines, inhibition of PAR-1 resulted in a significant reduction in tumor growth and metastatic lesions [135–137] (Figure 4).

Thrombin has also been targeted therapeutically. The direct oral thrombin inhibitor dabigatran has been reported to reduce breast cancer progression [138]. Dabigatran was also reported to act synergistically with cyclophosphamide to inhibit breast cancer cell growth and metastasis in vivo [139]. However, a recent study by Smeda et al. [140] reported that dabigatran promoted pulmonary metastasis in a murine model of breast cancer. Buijs et al. [141] also noted that it did not inhibit metastasis in mouse models. In a systematic review, Najidh et al. [142] concluded that direct oral anticoagulants (DOACs) do not affect tumor progression or metastasis in xenograft models. Some DOACs, however, have been observed to have antineoplastic and anti-inflammatory properties [143].

Heparins, polysaccharides belonging to the glycosaminoglycan family, bind ATIII and increase its inhibitory effect on thrombin and FX. They are known to induce a two- to four-fold increase in circulating TFPI, which has anti-angiogenic and anti-metastatic properties. Heparin and its derivatives have been shown to inhibit lung metastasis in breast cancer models. The low molecular weight (LMW) heparin tinzaparin has been shown to exhibit anti-metastatic properties in breast cancer cell lines [144]. Hirudin, a direct thrombin inhibitor, has also demonstrated anti-metastatic properties [145].

## Fibrinogen, D-dimer, and breast cancer

Fibrinogen molecules are approximately 45 nm in length and consist of two outer D-domains, each connected by a coiled-coil segment to a central E-domain [146]. It is a large, complex GP consisting of three pairs of polypeptide chains—Aα, Bβ, and γ—with molecular masses of 67, 57, and 47 kDa, respectively, for a total mass of about 340 kDa, including posttranslational carbohydrate additions [147]. Synthesized in the liver, fibrinogen plays a host of roles in human physiology, including blood clot formation, fibrinolysis, inflammation, wound healing, cellular and matrix interactions, and neoplasia.

D-dimer is a unique marker of fibrin degradation. It is a product of polymerized fibrin (cleaved fibrinogen catalyzed by FXIII) degraded by plasmin during fibrinolysis [148]. Unlike other FDPs that indicate only plasmin activation, D-dimer indicates activation of both thrombin and plasmin and is specific for coagulation and fibrinolysis. This unique quality has made D-dimer a valuable tool in various clinical scenarios, especially in VTE.

Fibrinogen and D-dimer are perhaps the most studied hemostatic markers in cancer, likely due to their ready availability and low cost. Several studies in cancer patients have shown that both fibrinogen and D-dimer are markers of cancer progression and prognostic indicators. Plasma D-dimer has been significantly associated with the clinical stage of breast cancer [149–154]. It also correlates significantly with histological stage [155, 156]. Kirwan et al. [19] established a significant association between elevated D-dimer levels and CTCs in metastatic breast cancer; likewise D-dimer has been shown to be a good marker for monitoring chemotherapy response in metastatic breast cancer [157]. This was validated in a previous study establishing D-dimer as a marker of metastasis and tumor progression [158]. These findings were reinforced by a recent retrospective study showing an association between CTCs and elevated D-dimer levels in patients with advanced breast cancer [159]. Thus, D-dimer is not only a marker of breast cancer progression but also a prognostic marker and a marker of breast cancer relapse [160–163].

While a cut-off value greater than 500 ng/mL is often considered indicative of VTE probability in the general population, in cancer patients, a value greater than 1,440 ng/mL is associated with an increased risk of VTE and has been incorporated into a risk assessment model [164]. Izuegbuna et al. [151] in similar research, calculated from a receiver operating characteristic (ROC) curve a sensitivity and specificity of D-dimer as a marker of lymph node involvement of 82.9% and 50%, respectively, with a quantitative value of 1,180 ng/mL indicating lymph node involvement. Gochhait et al. [165] also conducted a similar study but did not provide a quantitative value for lymph node involvement.

Fibrinogen, a known source of fibrin for tumor cells, has also been observed to be involved in the metastatic potential of tumor cells and their intravascular survival by preventing NK cell-mediated elimination [166]. Like D-dimer, fibrinogen is associated with breast cancer progression, clinical stage, tumor size, tumor stage, and lymph node involvement [167]. Cancer cells have been shown to form a fibrinogen-dependent bridge and transmigrate through the endothelium, highlighting the metastatic potential of fibrinogen [168]. Elevated fibrinogen levels have also been associated with OS in breast cancer patients and serve as a predictor for clinical response in patients undergoing chemotherapy [169–174]. Elevated fibrinogen also impairs treatment response to trastuzumab, with a cut-off value of > 2.88 g/L. Izuegbuna et al. [151] also showed via ROC analysis that a fibrinogen value greater than 4.47 g/L was associated with lymph node involvement.

## The fibrinolytic pathway and breast cancer

Fibrinolysis is a regulated enzymatic process that breaks down fibrin clots to prevent their intravascular accumulation and propagation, which can lead to organ ischemia and infarction. This process is essential for maintaining hemostatic balance. The role of fibrinolysis in cancer has been recognized for decades, with early 20th-century evidence demonstrating fibrinolytic properties in malignant tissues. These properties are primarily attributed to PAs secreted by tumors. While PAs function normally in physiological conditions, their regulation is frequently disrupted in various cancers.

The fibrinolytic system comprises proteases and their inhibitors. Key components include the protease plasmin, its inactive precursor plasminogen, and the tPA and uPA that activate it [175]. The activation of plasminogen to plasmin by tPA or uPA leads to fibrin clot degradation into soluble FDPs. Beyond fibrinolysis, plasmin degrades extracellular matrix (ECM) components to facilitate tissue remodeling and cell migration, and activates growth factors, playing a key role in cancer invasion and metastasis. Plasmin activity is counterbalanced by its specific inhibitor, α2-antiplasmin, and the nonspecific protease inactivator, α2-macroglobulin. Conversely, the activities of tPA and uPA are regulated by PAI-1, PAI-2, and APC inhibitor (PAI-3) [175–177]. Notably, certain proteases of the contact pathway of blood coagulation, plasma kallikrein and coagulation FXIIa, can also activate plasminogen, albeit less efficiently than tPA or uPA.

In addition to tissue remodeling and cell migration, the fibrinolytic system is heavily implicated in the metastatic process in breast cancer. It contributes to inflammatory cell recruitment, cytokine modulation, tumour cell growth and survival, angiogenesis, immune response, and growth factor regulation [178, 179]. Specific components—uPA, uPA receptor (uPAR), and PAI-1—are strongly associated with breast cancer progression. While these factors do not disrupt normal growth, homozygous deletion of the plasminogen gene (*Plg*^−/−^) in animal models leads to fertility issues, growth retardation, and reduced survival compared to wild-type animals (*Plg*^+/+^) [180].

Although tPA is present in both normal and malignant tissues, uPA is found predominantly in malignancies, where it plays a major role in tissue remodeling. Consequently, uPA, along with uPAR and PAI-1, are considered effective prognostic markers and therapeutic targets in breast cancer. uPA is a 53 kDa serine protease initially expressed as an inactive single-chain zymogen, pro-uPA [181]. It undergoes a two-step cleavage by proteases such as plasmin, cathepsins, and human kallikrein type 2 to form a two-chain, HMW-uPA, which is further cleaved into a proteolytically active LMW-uPA that retains its plasminogen-activating function. uPA comprises three domains: an amino-terminal domain containing the uPAR binding site, a carboxy-terminal catalytic domain, and a kringle domain of unknown function [182, 183]. By converting plasminogen to plasmin, uPA initiates the degradation of various ECM proteins, including fibrin, laminin, fibronectin, and osteopontin (OPN) [184]. Plasmin also activates pro-MMPs into active MMPs, further promoting ECM remodeling [185]. Furthermore, plasmin cleaves pro-uPA to generate HMW-uPA, creating a positive feedback loop [186]. uPA is overexpressed in tumor cells compared to normal cells, facilitating tumor invasion and migration [187]. Elevated uPA levels correlate with traditional prognostic factors such as tumor size, grade, and CTCs, and are associated with significantly shorter OS and progression-free survival in breast cancer patients [188–191].

**Figure 5:**
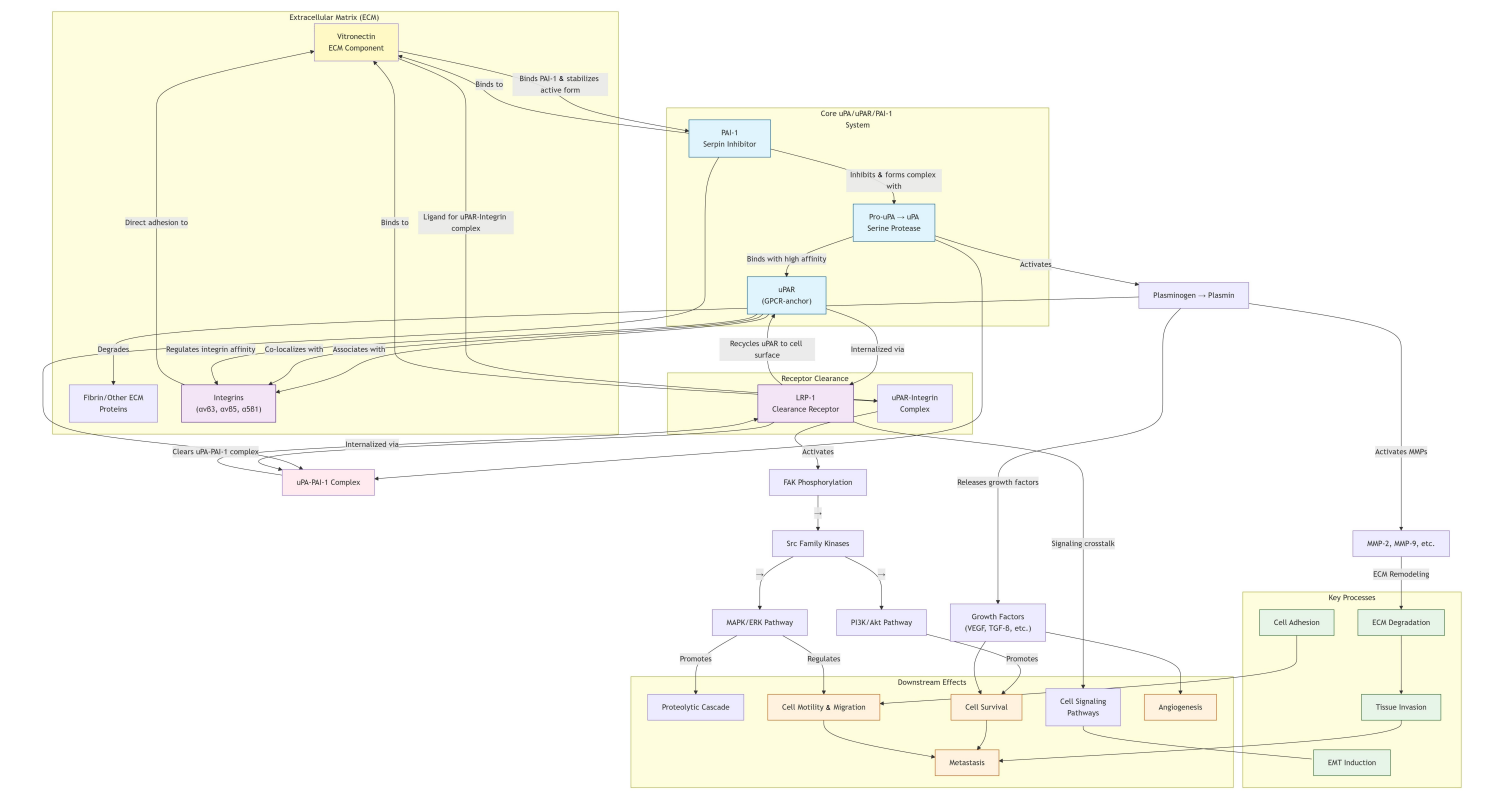
**The uPA, uPAR, and PAI-1 interaction in the fibrinolytic process promotes breast cancer progression.** EMT: epithelial-mesenchymal transition; GPCR: G protein-coupled receptor; LRP-1: lipoprotein receptor-related protein-1; MAPK: mitogen-activated protein kinase; MMP: matrix metalloproteinase; PAI-1: plasminogen activator inhibitor-1; PI3K: phosphatidylinositol 3-kinase; TGF-β: transforming growth factor-β; uPA: urokinase-type plasminogen activator; uPAR: urokinase-type plasminogen activator receptor; VEGF: vascular endothelial growth factor.

For plasmin to facilitate cancer cell migration via ECM degradation, uPA must bind to uPAR to initiate plasminogen activation. uPAR (CD87), a member of the Ly-6 superfamily, is a 55–60 kDa single-chain membrane GP receptor comprising approximately 313 amino acid residues [182, 192]. It consists of three domains (D1, D2, and D3) and is anchored to the outer cell membrane by a glycosylphosphatidylinositol moiety. While all three domains contribute to uPA binding, D2 and D3 are implicated in interactions with other proteins [193]. Beyond plasmin generation, uPAR influences cell adhesion and signaling by interacting with various cell surface proteins, including integrins (α5β1, α3β1, αvβ3, αvβ5), vitronectin, GPCRs, RTKs (e.g., EGF receptor and PDGF receptor), and the very low-density lipoprotein receptor [194–197]. These interactions activate signaling effectors such as focal adhesion kinase, Ras/MAPK, Rac1/MAPK, PI3K/AKT, and JAK1 pathways. Consequently, they trigger cellular responses—including migration, adhesion, EMT, proliferation, and angiogenesis—associated with breast cancer progression [198, 199] (Figure 5). uPAR is expressed at low levels under physiological conditions but is upregulated during inflammation and overexpressed in many tumors, including breast cancer [192]. This overexpression presents a prime opportunity for molecular imaging. For instance, recent reviews on the transformation of breast cancer diagnosis highlight the need for biomarkers that can guide emerging techniques like optical and hyperspectral imaging [200]. uPAR, with its specific upregulation on tumors, is an ideal molecular target for such advanced imaging modalities, potentially allowing for more precise tumor delineation and intraoperative margin assessment. Due to its low expression in healthy tissues and elevation in tumors, uPAR is a prime target for cancer diagnosis and therapeutic monitoring. uPAR-based imaging strategies to ascertain cancer aggressiveness are currently in clinical trials [201, 202]. This aligns with meta-analyses of computer-aided detection in breast cancer, which underscore the critical role of specific molecular targets in enhancing the accuracy and clinical utility of sophisticated imaging platforms like hyperspectral imaging [203]. Inhibition of uPAR expression prevents tumor invasion and migration; in breast cancer, uPAR inhibition alone or combined with trastuzumab blocked invasion and migration in various cell lines [204, 205]. uPAR is also linked to drug resistance; high uPAR expression reduces tamoxifen efficacy in patients [206] and confers resistance to tamoxifen in MCF-7 cells, and to doxorubicin and paclitaxel in MDA-MB-231 cells [207]. Thus, uPAR may serve as both a predictive factor for therapy response and a marker for breast cancer progression [208–211]. Integrating uPAR status with diagnostic imaging could therefore provide a more holistic view of tumor biology, potentially identifying not just the location of a tumor, but also its inherent aggressiveness and likelihood of treatment resistance.

PAI-1, a 45 kDa serine protease inhibitor and member of the SERPIN family (which includes PAI-2, PAI-3, protease nexin-1, and neuroserpin), is the principal inhibitor of uPA and tPA [212]. Paradoxically, while high PAI-1 expression is associated with metastasis and poor prognosis, PAI-2 appears to be protective [213]. Beyond inhibiting plasminogen activation, PAI-1 binds to vitronectin and low-density lipoprotein receptor-related protein-1 (LRP-1), augmenting cell motility and migration. LRP-1 facilitates the internalization of the uPA-PAI-1-uPAR complex via clathrin-mediated endocytosis. A high level of uPA-PAI-1 complex formation promotes breast cancer progression and predicts treatment response and survival [214, 215]. Multiple studies indicate that uPA/PAI-1 levels have good predictive value for response to certain chemotherapies in breast cancer [216–218], achieving level of evidence I for prognostic value in node-negative (pN0) patients and representing the only hemostatic breast cancer markers to reach this level [216]. Consequently, the American Society of Clinical Oncology recommended in 2007 the use of uPA/PAI-1 ELISA to assess recurrence risk in breast cancer patients [219, 220]. A large European Organisation for Research and Treatment of Cancer meta-analysis further validated the prognostic role of uPA and PAI-1 in breast cancer [221].

Given their critical roles, uPA, uPAR, and PAI-1 are attractive therapeutic targets. Several small molecules and novel inhibitors have been developed, with some undergoing clinical trials [222–228]. A recent study by Wrzeszcz et al. [229] suggested that a lower baseline plasma tPA antigen concentration and higher PAI-1 activity may be strong predictors of distant metastases and independent prognostic markers in breast cancer patients. The translation of these soluble biomarkers into clinically actionable intelligence is a key challenge. Future progress may lie in correlating these plasma biomarkers with non-invasive imaging signatures. As more research into breast cancer evolves, combining such data with advanced molecular imaging of targets like uPA [230] could enable more personalized, risk-stratified screening and diagnostic protocols.

## Endothelial cells and breast cancer

ECs, which form the single-cell layer lining blood vessels, are pivotal in vascular hemostasis, thrombosis, fibrinolysis, and vascular remodeling. The normal endothelium maintains an anticoagulant and antithrombotic state, but when compromised, it shifts to a procoagulant/prothrombotic phenotype. Normal ECs secrete substances like prostacyclin and nitric oxide that inhibit platelet aggregation, thus limiting thrombus extension [231]. They also express anticoagulants such as TFPI, thrombomodulin, endothelial protein C receptor, and heparin-like proteoglycans [232]. Upon activation, ECs participate in thrombin signaling via surface PAR-1 and, in response, thrombin induces the release of VWF, surface presentation of P-selectin, and production of platelet-activating factor (PAF) [233]. ECs contribute to fibrinolysis by releasing tPA and uPA, while also secreting PAI-1 to balance this activity in response to inflammatory cytokines [234]. Although ECs do not normally synthesize TF, activation by thrombin or inflammatory mediators can induce its expression [235]. Stored in Weibel-Palade bodies, VWF is mobilized upon vessel injury to mediate platelet adhesion to exposed ECM. In summary, ECs interact with multiple hemostatic markers that influence breast cancer progression.

ECs in breast cancer differ phenotypically and functionally from those in normal tissues [236]. The tumor microenvironment influences the EC phenotype, supporting tumor cell proliferation and migration. Breast cancer cells activate ECs and promote a niche that sustains survival, stemness, and metastasis [237]. In vitro, endothelial Jagged1 promotes breast cancer growth via Notch-dependent activation [238]. Markers of endothelial activation are also implicated in breast cancer progression. VWF, a large multimeric GP primarily secreted by ECs, is crucial in hemostasis, inflammation, angiogenesis, and cancer metastasis [239, 240]. Elevated VWF antigen levels are reported in various cancers, including breast cancer, with significantly higher levels in metastatic disease [241–243]. VWF is also a thrombotic risk factor in breast cancer. Pioneering research in the 1980s demonstrated that anti-VWF antibodies reduced platelet-cancer cell interactions in vitro and diminished metastatic potential in mice [244]. However, VWF’s activities may be cancer-type specific. In breast cancer, Dhami et al. [242] reported that cancer cells mediate EC activation, triggering the release of Weibel-Palade body contents (VWF, osteoprotegerin, angiopoietin-2), inducing angiogenesis and transendothelial migration, and promoting survival. VWF is also reported to shield metastatic cells from chemotherapy, though it can also exhibit anti-metastatic properties that could be therapeutically exploited [243]. Pre-surgical VWF levels in breast cancer patients correlate with tumor differentiation, grade, and predict disease relapse, suggesting its utility as a predictive factor [245].

PAF, another mediator synthesized by ECs (as well as neutrophils, platelets, and monocytes), is involved in angiogenesis, thrombosis, carcinogenesis, and metastasis [246]. In breast cancer, more malignant cells exhibit a greater capacity to synthesize and release PAF, metastasize, and express more PAF receptors on their membranes [247]. PAF may contribute to breast cancer initiation and promotion by enhancing cancer cell migration via phosphoinositide 3-kinase and/or Jun N-terminal kinase pathways, independently of the MAPK pathway [248].

## Future perspective

The ongoing re-evaluation of haemostatic proteins as major drivers of cancer progression, especially breast cancer has opened a transformative frontier for further evaluation of some haemostatic proteins as therapeutic targets.

The haemostatic protein TF, the primary initiator of the extrinsic coagulation cascade, is increasingly recognized as a critical player in cancer biology beyond its role in thrombosis. TF is aberrantly expressed on a variety of cancer cells and on tumor-associated endothelium, where it contributes to multiple hallmarks of cancer, including sustained proliferation, evasion of cell death, angiogenesis, and activation of invasion and metastasis [89, 97]. The strategic targeting of TF or its associated proteolytic pathway represents a promising therapeutic avenue in oncology.

The uPA system, a key fibrinolytic component, is intricately linked with TF and cancer progression. uPA and uPAR facilitate ECM degradation, cell migration, and invasion. The investigational oral uPA inhibitor, WX-671 (upamostat), has been evaluated in clinical trials. A randomized double-blind phase 1b study investigated WX-671 in combination with capecitabine versus capecitabine monotherapy in first-line human EGF receptor 2 (HER2)-negative metastatic breast cancer, demonstrating the clinical feasibility of this approach [249]. Similarly, a phase II proof-of-concept study in patients with non-resectable, locally advanced pancreatic cancer compared upamostat combined with gemcitabine versus gemcitabine alone. This study provided evidence of a signal for efficacy, supporting further investigation of uPA inhibition [250]. The clinical interest in this pathway is underscored by the FDA’s grant of Orphan Drug Designation for Mesupron (upamostat) for the treatment of pancreatic cancer in 2017 [251]. More recently, a phase I trial confirmed the safety and tolerability of upamostat combined with gemcitabine in patients with locally unresectable or metastatic pancreatic cancer, but without significant tumor response, suggesting the need for further investigation [252]. Demonstrating unequivocal efficacy is very important to move it forward. Thus, future trials must clearly define the biological context (e.g., high uPA/uPAR tumor expression) and identify synergistic combination partners to move beyond proof-of-concept.

Beyond the uPA system, TF itself has emerged as a potent and specific therapeutic target, and this is because of it being the primary initiator of the extrinsic coagulation cascade, as well as being aberrantly expressed on a variety of cancer cells and on tumor-associated endothelium, where it contributes to multiple hallmarks of cancer, including sustained proliferation, evasion of cell death, angiogenesis, and activation of invasion and metastasis. TV, an ADC targeting TF and delivering the cytotoxic payload MMAE, has demonstrated significant clinical success. The pivotal study published in the New England Journal of Medicine established TV as an effective second- or third-line therapy for recurrent cervical cancer, leading to its regulatory approval [253]. This was built upon the earlier first-in-human, phase 1–2 InnovaTV 201 trial, which showed promising antitumor activity of TV across a range of advanced or metastatic solid tumours [254]. The clinical development of TV continues to expand, with numerous trials registered in databases such as the EU Clinical Trials Register, exploring its utility in various other malignancies [255]. The immediate future of TF driven oncology is through the clinical validation and expansion of the targeted biologics, TV, as well as development of similar TF ADCs to tackle other TF-positive solid tumors, such as ovarian, breast, bladder, and squamous non-small cell lung cancers, as indicated by ongoing phase II/III trials. However, managing class-specific ADC toxicities such as ocular adverse events (conjunctivitis, dry eye) and peripheral neuropathy is important to improve patients’ quality of life and therapeutic sustainability.

The therapeutic targeting of TF is rapidly diversifying beyond ADCs. Immunotherapeutic approaches are now at the forefront of research. Recent work has demonstrated the conversion of anti-TF antibody sequences into novel formats, including chimeric antigen receptors (CARs) and bispecific T cell engagers (BiTEs) [256]. Preclinical studies have shown that CAR-T cells modified to target TF can potently inhibit the growth and metastasis of established TF-positive tumors in murine models [257]. The foundational concept of targeting TF with an ADC, as demonstrated by Breij et al. [258], showed that such a conjugate exhibits potent therapeutic activity against a broad range of solid tumors, validating TF as a robust target for drug delivery. This has been extended to bispecific antibodies; for instance, Pan et al. [259] characterized a novel bispecific antibody that engages T cells to target TF-positive tumors. Most recently, a novel TF-targeting BiTE molecule was shown to provide effective targeting and potent cytotoxicity against cervical cancer cell lines, highlighting the translational potential of this modality [260].

Furthermore, TF’s role extends beyond therapy to cancer diagnostics and understanding treatment resistance. It has been investigated as a novel diagnostic target for the early detection of ovarian cancer using targeted ultrasound microbubbles [261]. In glioblastoma, TF has been identified as a critical regulator of tumor remodeling and pro-tumorigenic processes induced by radiation therapy [262]. This positions TF at the crossroads of coagulation and the cellular response to a key cancer treatment modality, suggesting that its inhibition could modulate the tumor microenvironment to improve therapeutic outcomes [263]. The successes of TF-targeted CAR-T cells and BiTEs at the preclinical stage are a paradigm shift towards the long-term potentials of haemostasis-driven oncology. These strategies can be envisaged where a TF-BiTE could help redirect endogenous T cells to lyse TF-positive tumor cells and ECs simultaneously, disrupting the tumor’s blood supply. However, some off-target activity can be a major concern as TF is constitutively expressed at low levels in critical epithelial (skin, gut) and vascular compartments. Thus, CAR-T/BiTE therapies must be engineered for exquisite tumor selectivity, potentially through logic-gated systems (requiring a second tumor antigen) or tuning of affinity to spare low-TF normal tissues. Despite these things, adverse events like the cytokine release syndrome, the immune effector cell-associated neurotoxicity syndrome and the T cell exhaustion in the solid tumor microenvironment are important immunological concerns that should also be tackled.

The interplay between coagulation proteases and cancer biology extends beyond initial thrombotic events to directly influence key drivers of tumor progression and metastatic dissemination. Central to this interplay is the serine protease thrombin, which, through the cleavage of specific substrates, activates a cascade of pro-tumorigenic pathways.

Direct thrombin inhibition has demonstrated significant anti-cancer effects. A recent study showed that recombinant tyrosine-sulfated haemathrin, a novel thrombin inhibitor, effectively suppresses cancer cell migration and invasion in vitro [264]. This aligns with earlier findings that thrombin is a critical therapeutic target for non-small-cell lung cancer, where its inhibition disrupts the formation of vasculogenic mimicry, a mechanism by which aggressive tumors form fluid-conducting channels independent of ECs [265]. The regulatory scope of thrombin includes the cleavage of key receptors in the tumor microenvironment. A 2025 study revealed that thrombin cleaves membrane-bound endoglin, a TGF-β co-receptor, an action that may contribute to pathological signaling and highlights a novel mechanism by which thrombin can modulate angiogenesis and cellular responses [266].

Endoglin (CD105) itself is a well-established marker of tumor angiogenesis, and its targeting has been an area of active investigation, with lessons learned from past attempts informing current strategies [267]. Promisingly, a human endoglin-CD3 BiTE antibody has been developed, demonstrating a potent anti-tumor effect in vivo by redirecting T cells to the tumor vasculature [268]. Thus, endoglin (CD105)-targeted BiTEs could offer a strategy to specifically target the proliferating tumor vasculature. Furthermore, due to its specific expression on proliferating ECs, endoglin serves as a valuable target not only for therapy but also for the molecular imaging of cancer, as highlighted in a systematic review [269].

A critically important thrombin substrate in oncology is the matricellular protein OPN. Thrombin cleavage of OPN is a pivotal event that initiates its tumor-promoting activity [270]. This mechanistic insight is strongly supported by in vivo evidence showing that anti-OPN antibodies, which mimic the effect of blocking thrombin-cleaved OPN, replicate a tumor suppression phenotype in knock-in mice engineered with a thrombin cleavage-resistant form of the protein [271]. The functional importance of this thrombin cleavage domain in OPN has been established for over a decade, with studies showing that its deletion impairs breast cancer cell adhesion, proteolytic activity, tumorigenicity, and metastasis [272]. The consequences of thrombin cleavage extend to modulating the host anti-tumor immune response, further cementing the thrombin-OPN axis as a critical pathway in oncology [273]. The broader role of OPN in cancer and its potential as a therapeutic target have been extensively reviewed, underscoring its significance across multiple cancer types [274].

Beyond the thrombin-OPN axis, other haemostatic proteins contribute to the malignant phenotype. The transmembrane GP podoplanin (PDPN), a known activator of platelet aggregation, is a potential therapeutic target for thrombotic complications in cancer patients [275]. Clinically, PDPN expression in cancer-associated fibroblasts has been identified as an independent predictor of unfavorable prognosis in node-negative, hormone receptor-positive/HER2-negative breast cancer patients, linking it directly to disease outcomes [276]. Therapeutically, a cancer-specific anti-PDPN monoclonal antibody, PMab-117-mG2a, has shown promising antitumor activities in human tumor xenograft models, validating PDPN as a viable target for antibody-based therapy [277]. In all of these things, the central dilemma is how to separate anticancer effects from haemostasis disruption. The direct and chronic thrombin inhibition for cancer poses a significant bleeding risk. The solution lies in developing “biased inhibitors” that block thrombin’s signaling via PARs while sparing its fibrin-generating function, or in creating non-absorbable agents that act locally within the tumor microenvironment.

The role of aspirin, an antiplatelet and a cyclo-oxygenase (COX) inhibitor in cancer control cannot be overemphasized. Recent works have shown that post-diagnostic aspirin use was associated with a lower breast-cancer-specific mortality [278–280] and breast cancer recurrence [281]. In colorectal cancer (CRC), the use of aspirin has been linked to a lower risk of the disease [282–284]. It has also been linked to a significant cancer-specific survival (though not OS) in users with CRC compared to non-users, preventing progression to stage IV disease [285]. However, the phase 3 aspirin after completion of standard adjuvant therapy for CRC trial showed that it had no significant effect on DFS and OS [286]. Studies have also shown that aspirin can function as an adjunct immunotherapy through the regulation of the gut microbiota and certain immune checkpoints [287–289] and also preventing metastasis by enhancing T cell immunity [290]. These studies show that an antiplatelet agent like aspirin can influence the tumor milieu and help the body fight cancer. Like aspirin, celecoxib, a specific COX-2 inhibitor, did not improve DFS in stage 3 CRC patients [291]; but a sub-analysis of the same trial (NCT01150045) showed there was significant OS in those with *PIK3CA* mutational status [292]. In the same vein, the Randomized European Celecoxib Trial, a phase 3, randomized, double-blind study (NCT02429427), did not show any evidence of DFS benefit in breast cancer patients [293]. However, a subgroup analysis of 655 breast cancer patients from the study that were not on chemotherapy reduced the hazard ratio (HR) for recurrence over 10 years by 35% (HR = 0.65, 95% confidence interval 0.41–1.04, *P* = 0.035) [294], making celecoxib a viable option as an adjunct therapy. Currently, celecoxib is being investigated to determine its efficacy in the inhibition of radiotherapy-induced cytokine release associated with metastasis development in triple-negative breast cancer patients (NCT07104266) [295].

In summary, haemostatic proteins are integral to the cancer phenotype. As comprehensively discussed in a recent 2025 review, thrombin plays a driving role in the paradoxical interplay between cancer metastasis and the vascular system [296]. Its actions, from the direct inhibition of cell migration to the proteolytic activation of key substrates like endoglin and OPN, create a permissive environment for tumor growth and spread. In the same vein, the haemostatic proteins TF and uPA are not merely incidental features of the tumor microenvironment but are active drivers of cancer progression and metastasis. The successful clinical development of agents like TV and the ongoing investigation of upamostat, along with the promising preclinical data for CAR-T and BiTE therapies, firmly establish the targeting of haemostatic proteins as a valid and potent strategy in the oncological armamentarium. The continued development of targeted therapies against these proteins and their pathways holds significant promise for novel anti-cancer strategies.

## Conclusions

Recent decades have significantly advanced our understanding of hemostatic markers in breast cancer. Activation of the hemostatic system is common in cancer, and many hemostatic markers are implicated in cancer cell proliferation and migration (Table 2). These markers often correlate with increased tumor burden and poor prognosis. Although clinical studies suggest that hemostatic markers are suitable for monitoring breast cancer progression, validation remains a challenge. To date, only uPA and PAI-1 have been validated as prognostic markers. Others, such as fibrinogen and D-dimer, show great promise; D-dimer is already validated for assessing thrombosis risk and the potential need for thromboprophylaxis in cancer patients.

**Table 2:** Role of hemostatic proteins in breast cancer progression.

**Hemostatic protein**	**Role in breast cancer**
Tissue factor	Cell migration, anti-apoptosis, angiogenesis
Thrombin	Cell extravasation, angiogenesis
Fibrinogen	Cell extravasation, lymph node metastasis
D-dimer	Cell extravasation, lymph node metastasis
Urokinase-type plasminogen activator receptor	Cell adhesion, cell migration, angiogenesi
Urokinase-type plasminogen activator	Cell adhesion, cell migration, angiogenesis
Plasminogen activator inhibitor-1	Cell migration, cancer relapse
Von Willebrand factor	Cell metastasis, angiogenesis, cancer relapse
Platelet-activating factor	Cell metastasis, angiogenesis

These developments underscore the diagnostic and therapeutic potential of hemostatic markers. A TF-antibody conjugate has been developed, and therapies targeting uPA and uPAR are in clinical development. After proper validation, hemostatic markers could serve as valuable diagnostic and therapeutic tools, potentially reducing morbidity and mortality in breast cancer and other malignancies. The ongoing HYPERCAN study, a prospective, multicenter, observational study initiated in 2012, aims to assess whether a hypercoagulable state can predict cancer diagnosis in healthy individuals or disease recurrence, progression, and thrombosis in cancer patients. It is evaluating several biomarkers, including D-dimer, fibrinogen, thrombin generation, TF, F1+2, microparticle procoagulant activity, protein C, protein S, tPA, PAI-1, FVIII, and FXIII. It is hoped that such studies will yield clinically useful results. For now, further clinical studies are required to validate these hemostatic biomarkers and translate them into routine clinical practice. In addition, accelerating the translation of some of these biomarkers through biomarker validation and standardization, and understanding context-dependent duality (haemostatic proteins have paradoxical roles. PDPN’s role in thrombosis must be balanced against its potential activity in immune regulation) can improve the trajectory of haemostasis-driven oncology through the development of specific inhibitors that disarm the tumor’s haemostatic toolkit for invasion and survival. Realizing these potential mandates, a concerted effort to solve the critical challenges of toxicity management, biomarker development, and context-aware drug design. Success in this endeavor will not only provide new weapons against cancer but will also offer a unique paradigm for targeting the tumor microenvironment itself.

## Abbreviations

ADC: antibody-drug conjugate
APC: activated protein C
asTF: alternatively spliced tissue factor
BiTEs: bispecific T cell engagers
CARs: chimeric antigen receptors
COX: cyclo-oxygenase
CRC: colorectal cancer
CTCs: circulating tumor cells
DFS: disease-free survival
DOACs: direct oral anticoagulants
EC: endothelial cell
ECM: extracellular matrix
EGF: epidermal growth factor
EMT: epithelial-mesenchymal transition
FDPs: fibrin degradation products
FVII: factor VII
FVIIa: activated factor VII
GP: glycoprotein
GPCRs: G protein-coupled receptors
HER2: human epidermal growth factor receptor 2
HMWK: high molecular weight kininogen
HR: hazard ratio
JAK: Janus-associated kinase
LMW: low molecular weight
LRP-1: lipoprotein receptor-related protein-1
MAPK: mitogen-activated protein kinases
MHC: major histocompatibility complex
MMAE: monomethyl auristatin E
MMPs: matrix metalloproteinases
mTOR: mammalian target of rapamycin
NK: natural killer
OPN: osteopontin
OS: overall survival
PAF: platelet-activating factor
PAI-1: plasminogen activator inhibitor-1
PARs: protease-activated receptors
PAs: plasminogen activators
PDGF: platelet-derived growth factor
PDPN: podoplanin
PI3K: phosphatidylinositol 3-kinase
ROC: receiver operating characteristic
RTK: receptor tyrosine kinase
STAT: signal transducer and activator of transcription
TAFI: fibrinolysis inhibitor
TCIPA: tumor cell-induced platelet aggregation
TF: tissue factor
TFPI: tissue factor pathway inhibitor
TGF-β: transforming growth factor-β
tPA: tissue-type plasminogen activator
TV: tisotumab vedotin
uPA: urokinase-type plasminogen activator
uPAR: urokinase-type plasminogen activator receptor
VEGF: vascular endothelial growth factor
VTE: venous thromboembolism
VWF: von Willebrand factor

## Declarations

### Copyright

© The Author(s) 2026.
